# Ventilator‐induced pulse pressure variation in neonates

**DOI:** 10.14814/phy2.12716

**Published:** 2016-02-23

**Authors:** Linda Heskamp, Benno Lansdorp, Jeroen Hopman, Joris Lemson, Willem‐Pieter de Boode

**Affiliations:** ^1^ Department of NeonatologyRadboudum Amalia Children's HospitalNijmegenThe Netherlands; ^2^ MIRA‐Institute for Biomedical Technology and of Technical Medicine, of the Faculty of Science and TechnologyUniversity of TwenteEnschedeThe Netherlands; ^3^ Department of Intensive Care MedicineRadboud university medical centreNijmegenThe Netherlands; ^4^ Department of RadiologyRadboud university medical centreNijmegenThe Netherlands

**Keywords:** Arterial blood pressure variability, fluid responsiveness, neonatal hemodynamics, neonates, pulse pressure variation

## Abstract

During positive pressure ventilation, arterial pressure variations, like the pulse pressure variation (PPV), are observed in neonates. However, the frequency of the PPV does not always correspond with the respiratory rate. It is hypothesized that PPV is caused by cardiopulmonary interaction, but that this mismatch is related to the low respiratory rate/heart rate ratio. Therefore, the goal of this study is to investigate the relation between PPV and ventilation in neonates. A prospective observational cross‐sectional study was carried out in a third‐level neonatal intensive care unit in a university hospital. Neonates on synchronized intermittent mandatory ventilation (SIMV) or high‐frequency ventilation (HFV) participated in the study. The arterial blood pressure was continuously monitored in 20 neonates on SIMV and 10 neonates on HFV. In neonates on SIMV the CO_2_ waveform and neonates on HFV the thorax impedance waveform were continuously monitored and defined as the respiratory signal. Correlation and coherence between the respiratory signal and pulse pressure were determined. The correlation between the respiratory signal and pulse pressure was ‐0.64 ± 0.18 and 0.55 ± 0.16 and coherence at the respiratory frequency was 0.95 ± 0.11 and 0.76 ± 0.4 for SIMV and HFV, respectively. The arterial pressure variations observed in neonates on SIMV or HFV are related to cardiopulmonary interaction. Despite this relation, it is not likely that PPV will reliably predict fluid responsiveness in neonates due to physiological aliasing.

## Introduction

To ensure adequate end‐organ perfusion and tissue oxygenation in neonates an adequate filling pressure (preload) is necessary (Evans 2003). Therefore, in case of true hypovolemia, a fluid bolus can be life saving. However, volume expansion in an already hypervolemic neonate is associated with an adverse neurological outcome (Greenough et al. 2002), increased prevalence of chronic lung disease (Van Marter et al. 1992), and increased mortality (Ewer et al. 2003). To avoid these adverse effects it is important to determine if a neonate will increase stroke volume when a fluid bolus is given, that is, will be fluid responsive. Studies show that currently used clinical and static hemodynamic parameters are not able to accurately predict fluid responsiveness in neonates (Evans 2003; de Boode 2010). In adults, it is shown that dynamic indices based on preload changes secondary to cardiopulmonary interaction during positive pressure ventilation can predict fluid responsiveness (Michard et al. 2000; Feissel et al. 2001; Michard and Teboul 2002). According to the Frank–Starling relation, these ventilator‐induced preload changes result in variation in stroke volume (Jardin et al. 1983; Michard 2005; Cannesson et al. 2011) and the proposed surrogate of stroke volume, the arterial pulse pressure (PP) (Jardin et al. 1983). Marik et al. (2009) reported in a meta‐analysis that pulse pressure variation (PPV) predicts fluid responsiveness with a sensitivity of 89% and specificity of 88% in the adult population under specific conditions.

In clinical practice PPV is also observed in neonates. However, the frequency of these variations does not always correspond with the respiratory rate (RR). The observed mismatch in frequencies in neonates might be explained by the lower heart rate to respiratory rate (HR/RR) ratio observed in neonates compared to adults (due to a relative higher basal RR than heart rate [HR]). Also in adult patients, it has been shown that the absolute and predictive value of PPV is diminished when the HR/RR ratio is lower than 3.6 (De Backer et al. 2009). In neonates the HR/RR ratio is in general less than 3.6. Because of this observed mismatch, it is important to first determine whether the observed PPV in neonates is indeed related to cardiopulmonary interaction before investigating the ability of PPV to predict fluid responsiveness in neonates. To our knowledge, there are no studies that demonstrated this relation. Therefore, the goal of this study is to investigate the relation between PPV and cardiopulmonary interaction in neonates ventilated with the two most common modes of ventilation, that is, synchronized intermittent mandatory ventilation (SIMV) and high‐frequency ventilation (HFV).

## Materials and Methods

### Study population

This study was performed in (preterm) neonates admitted to the neonatology intensive care unit of the Radboud University Medical Center. The hospital ethics committee waived the need for informed consent. Inclusion criteria were the presence of an intra‐arterial catheter and mechanical ventilation with SIMV or HFV (Leoni Plus^®^, Heinen & Löwenstein, Bad Ems, Germany), and absence of spontaneous breathing activity.

### Data acquisition

Invasive ABP was continuously measured using an arterial catheter. In patient on SIMV, the CO_2_ waveform was continuously measured using the capnograph and in patients on HFV, the thoracic impedance waveform was continuously measured. The CO_2_ waveform or thoracic impedance waveform were defined as Resp. ABP and Resp were acquired via the IntelliVue Patient Monitor (MP90^®^, Philips Healthcare, Best, The Netherlands) and recorded with Trendface^®^ (ixellence GmbH, Wildau, Germany) with a sampling frequency of 62.5 Hz.

### Data analysis

**Figure 1:**
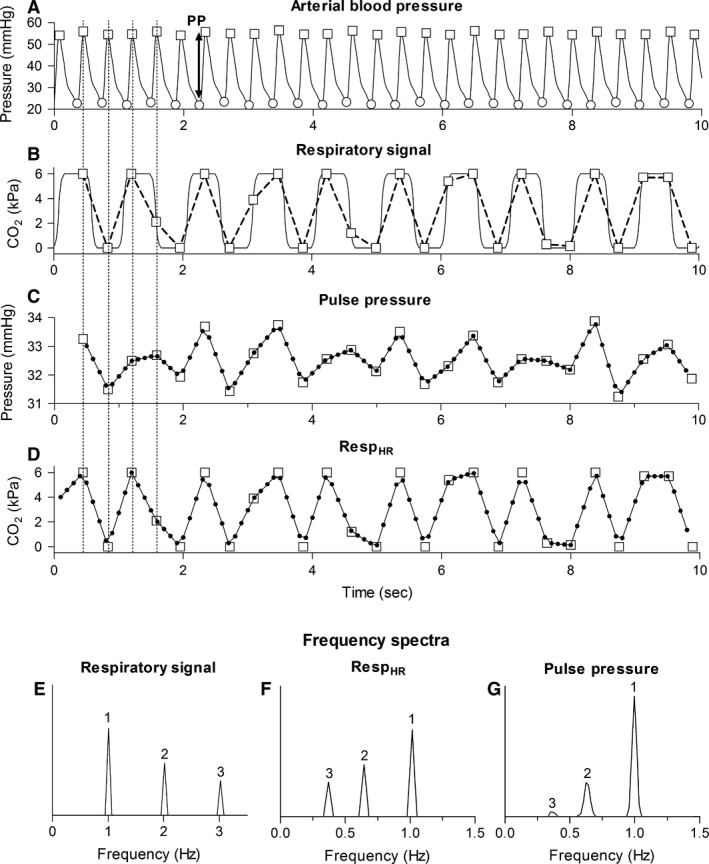
Representation of the steps performed during the data analysis (HR = 2.7 Hz, SIMV with RR = 1 Hz). (A) Arterial blood pressure with systolic (squares) and diastolic blood pressure (circles). (B) Respiratory signal (CO_2_, solid line), respiratory signal values obtained at the moment that the systolic pressure is reached (squares) and Resp_HR_ (dashed line). (C) Interpolated PP signal. The squares depict PP at the moment systolic pressure is reached. (D) Interpolated Resp_HR_. The squares depict the respiratory value at the moment systolic pressure was reached. (E) Frequency spectrum of the respiratory signal containing: RR (1), 2RR (2), and 3RR (3). (F, G) Frequency spectrum of Resp_HR_ and PP containing RR (1) and the alias frequencies of 2RR and 3RR (2 and 3, respectively). HR, heart rate; RR, respiratory rate; PP, pulse pressure; Resp_HR_, the respiratory values at the moment systolic pressure was reached.

Two minutes of recording without artifacts in ABP and Resp were manually selected for further processing and analysis. The systolic and diastolic pressures were detected using a peak detection algorithm in Matlab (Matlab R2011A^®^, MathWorks Inc., Natick, MA). PP was defined as the difference between the systolic and the preceding diastolic pressure, as depicted in Figure 1A. In order to simulate the effect that the respiratory cycle is sampled with the heart rate (because the effect of the ventilation on the circulation is expressed per heartbeat) and to determine whether the PPV is caused by cardiopulmonary interaction, the value of the respiratory signal was taken at the moment the systolic pressure of a heartbeat was reached, as illustrated in Figure 1A and B by Resp_HR_.

### Frequency spectra

Frequency spectra were obtained from Resp, Resp_HR_, and PP with a Fourier Transform. However, the Fourier Transform can only be applied if the time series have evenly spaced samples. Because PP and Resp_HR_ have an irregular sampling frequency, namely heart rate, in both time series the samples are unevenly spaced. To obtain evenly spaced time series, PP and Resp_HR_ were linear interpolated and resampled at 10 Hz (Fig. 1C and D). Frequency spectra were obtained using Welch's method with a 30‐sec Hamming window and 50% overlap (Fig. 1E, F and G).

### Effect of the low HR/RR ratio

#### Principle of aliasing

**Figure 2:**
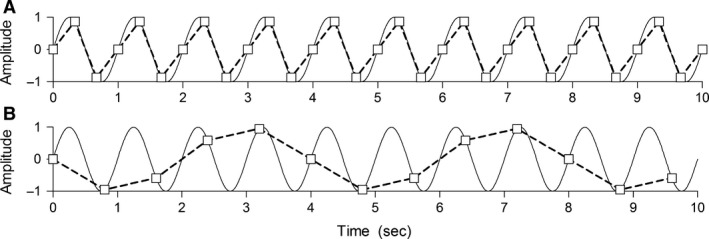
Aliasing: the solid line is a sinusoidal waveform of 1 Hz and represents the respiratory signal; the squares are the obtained samples (representing the heartbeats). The dashed line is the reconstructed sinusoidal waveform (based on the squares), representing Resp_HR_. (A) RR =1 Hz and HR = 3 Hz, the respiratory signal is adequately represented as sinusoidal waveform of 1 Hz. (B) RR = 1 Hz and HR = 1.25 Hz, the sinusoidal waveform is undersampled which results in a reconstructed sinusoidal waveform with a lower frequency, in this case 0.25 Hz (the alias frequency). RR, respiratory rate; HR, heart rate.

It is hypothesized that the observed mismatch in frequencies in neonates can be explained by the HR/RR ratio seen in neonates. This is because at least two samples (in this case heartbeats) per respiratory cycle are needed to adequately represent the respiratory signal and to avoid undersampling [Nyquist–Shannon sampling theorem (Jerri 1977)]. As depicted in Figure 2A, if there are, for example, three heartbeats per respiratory cycle, the respiratory signal is adequately represented as a sinusoidal waveform of 1 Hz. However, when there are less than two heartbeats per respiratory cycle, undersampling occurs, wherefore the respiratory signal is not represented as the original sinusoidal waveform of 1 Hz, but as a sinusoidal waveform with a lower frequency (Fig. 2B). This phenomenon is called aliasing and this lower frequency is called the alias frequency. Hence, if the respiratory signal is undersampled due to a HR/RR ratio below 2, Resp_HR_ will not vary with RR, but the so called alias frequency of RR.

#### Aliasing in HFV

During HFV, the HR/RR ratio is always smaller than 2. This indicates that aliasing of RR will occur. RR_alias_ can be predicted using the following equation with *n* the closest integer multiple of HR to RR.

$${\text{RR}}_{\mathrm{alias}}=\left|\text{nHR}-\text{RR}\right|$$

RR_alias_ was compared to the observed frequency in Resp_HR_ and PP. If PP varies with RR_alias_, this will support the hypothesis that in neonates on HFV, PPV is caused by cardiopulmonary interaction and that the mismatch between the frequency in PP and RR is due to the HR/RR ratio smaller than 2.

#### Aliasing in SIMV

Generally, in patients ventilated with SIMV without spontaneous breathing activity the HR/RR ratio is not smaller than 2. Hence, aliasing of RR will not occur. However, Resp is not a pure sinusoidal waveform. Therefore, Resp does not only contain the fundamental frequency RR, but also higher frequencies from which the multiples of RR (2RR, 3RR, etc.) are the most prominent (Fig. 1E). The ratio between HR and these higher frequencies (e.g., 2RR and 3RR) can be smaller than 2. Therefore, aliasing still occurs even with a HR/RR ratio larger than 2. This means, when sampling Resp with HR, it is expected that in the frequency spectrum of Resp_HR_ alias frequencies of these higher frequencies (e.g., alias frequencies of 2RR and 3RR) will be observed (Fig. 1F). If these alias frequencies are also observed in PP, as illustrated in Figure 1G, this will support the hypothesis that in neonates on SIMV PPV is caused by cardiopulmonary interaction and that the presence of frequencies in PP not equal to RR is caused by the low HR/RR ratio.

#### Relation between ventilation and PPV

The relation between ventilation and PP was determined in both the time domain and frequency domain. In the time domain, the Spearman correlation was determined between PP and Resp_HR_, while correcting for the time delay between PP and Resp_HR_. For SIMV, the maximum negative correlation was selected because during inspiration Resp decreases (CO_2_ decreases) while PP increases. For HFV, the maximum positive correlation was selected because during inspiration Resp increases (thoracic impedance increases) while PP increases. In the frequency domain, the coherence function was determined between PP and Resp_HR_. Briefly, the coherence function is the correlation determined for each frequency region. The coherence function ranges between 0 and 1 (no relation and perfect relation, respectively). The coherence value was determined at RR for SIMV and the observed alias frequency in Resp_HR_ for HFV. A relation between ventilation and PP was presumed when coherence reached the cut‐off value for significant coherence. The cut‐off value was 0.42 for SIMV and 0.46 for HFV (Faes et al. 2004).

## Results

### Patient characteristics

For SIMV, 20 (10 males) patients were included with a median gestational age of 33 weeks (IQR 29–39 weeks), a median postnatal age of 8 days (IQR 2–13 days), and a median birth weight of 1745 g(IQR 1119–3238 g). For HFV, 10 (7 males) patients were included with a median gestational age of 27 weeks (IQR 25–34 weeks), a median postnatal age of 16 days (IQR 10–21 days), and a median birth weight of 1035 g(IQR 819–2400 g). The position of the arterial catheter varied between the left and right radial and tibial artery and the umbilical artery. The HR/RR ratio was 2.6 ± 0.5 and 0.4 ± 0.1 for neonates on SIMV and HFV, respectively.

### HFV

#### Time domain and frequency domain

**Figure 3:**
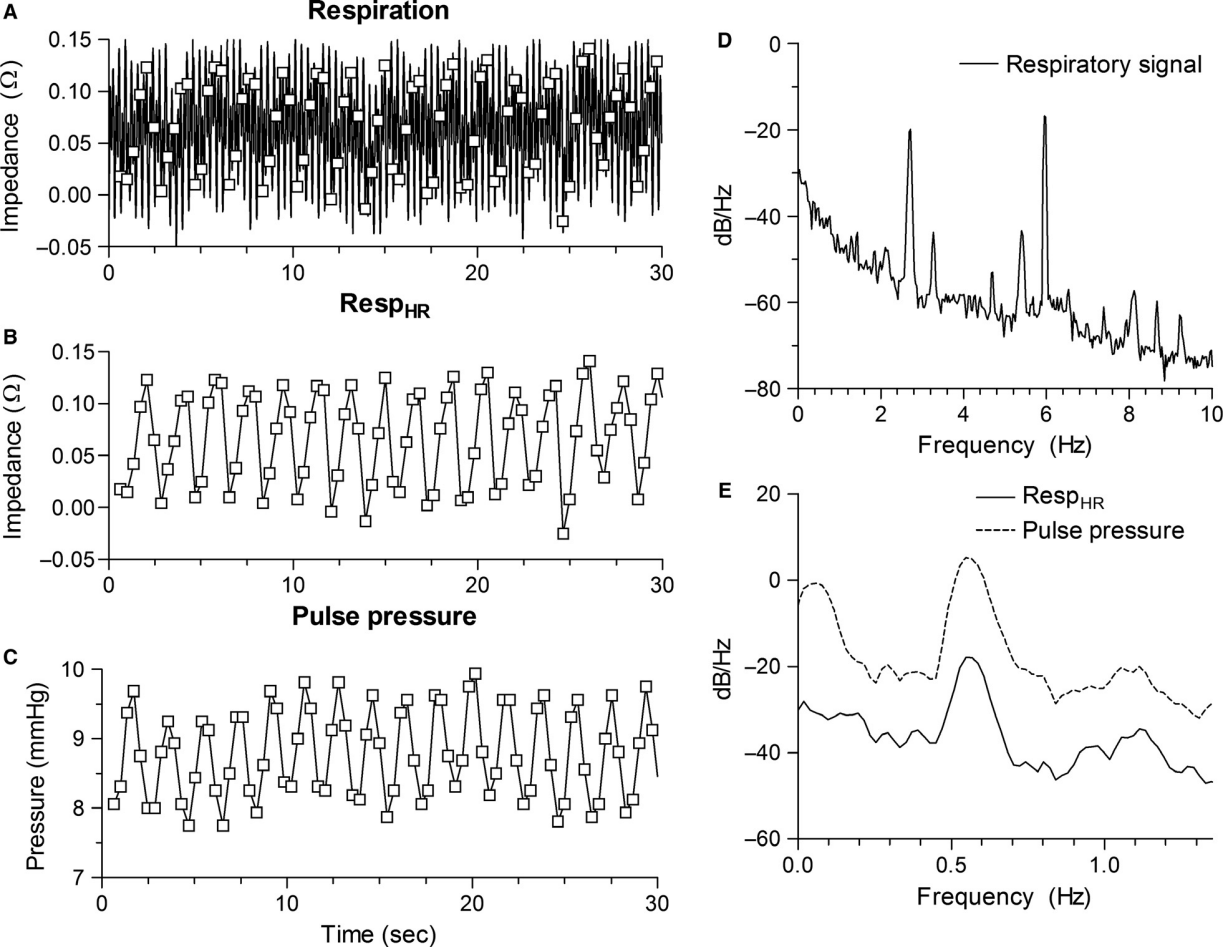
Respiratory signal, Resp_HR_, and pulse pressure in the time and frequency domain for an example patient ventilated with HFV (RR = 6 Hz, HR = 2.7 Hz). (A) Respiratory signal with time points that systolic pressure is reached (squares). (B) Resp_HR_. (C) Pulse pressure. (D) Frequency spectrum of the respiratory signal. (E) Frequency spectra of Resp_HR_ (solid) and pulse pressure (dashed). RR, respiratory rate; HR, heart rate; Resp_HR_, the respiratory values at the moment systolic pressure was reached.

Figure 3 depicts the respiratory signal (Resp), the values of the respiratory signal at the moment systolic pressure was reached (Resp_HR_) and PP in the time domain and frequency domain for a patient supported with HFV to illustrate the results (RR = 6 Hz, HR = 2.7 Hz). First, in the time domain PPV is observed (Fig. 3C). In the frequency domain it is observed that the frequency of this PPV equals the frequency observed in Resp_HR_ (Fig. 3E). Second, also a variation in PP with frequency <0.1 Hz is observed that is not observed in Resp_HR_, representing the Mayer waves (Fig. 3E). Mayer waves are oscillations in the arterial blood pressure that occur spontaneously and represent sympathetic activity (Julien 2006). The findings illustrated in Figure 3 are observed in all patients ventilated with HFV.

#### Aliasing

Table 1 shows the alias frequency of RR (RR_alias_), the frequency in Resp_HR_, and the frequency in PP for all patients ventilated with HFV. There is no difference observed between RR_alias_, the frequency in Resp_HR_, and the frequency in PP.

**Table 1:** Alias frequency of RR (RR_alias_) and the observed frequency in Resp_HR_ and pulse pressure for patients ventilated with HFV

Patient	HR/RR ratio	RR_alias_	Frequency in Resp_HR_	Frequency in PP
1	0.45	0.57	0.55	0.55
2	0.55	0.60	0.63	0.63
3	0.41	1.20	1.19	1.17
4	0.36	0.60	0.64	0.64
5	0.23	0.73	0.72	0.72
6	0.24	0.60	0.61	0.45
7	0.34	0.15	0.23	0.20
8	0.31	0.75	0.66	0.66
9	0.27	0.73	0.84	0.84
10	0.29	1.30	1.19	1.27

Resp_HR_, respiratory signal at the moment of systolic pressure is reached; PP, pulse pressure.

### Relation between ventilation and PPV

The Spearman correlation between Resp_HR_ and PP, while correcting for the time delay, was 0.55 ± 0.16. The coherence value between Resp and PP at RR_alias_ was 0.76 ± 0.4. Only in two patients the coherence value was not significant.

### SIMV

#### Time domain and frequency domain

**Figure 4:**
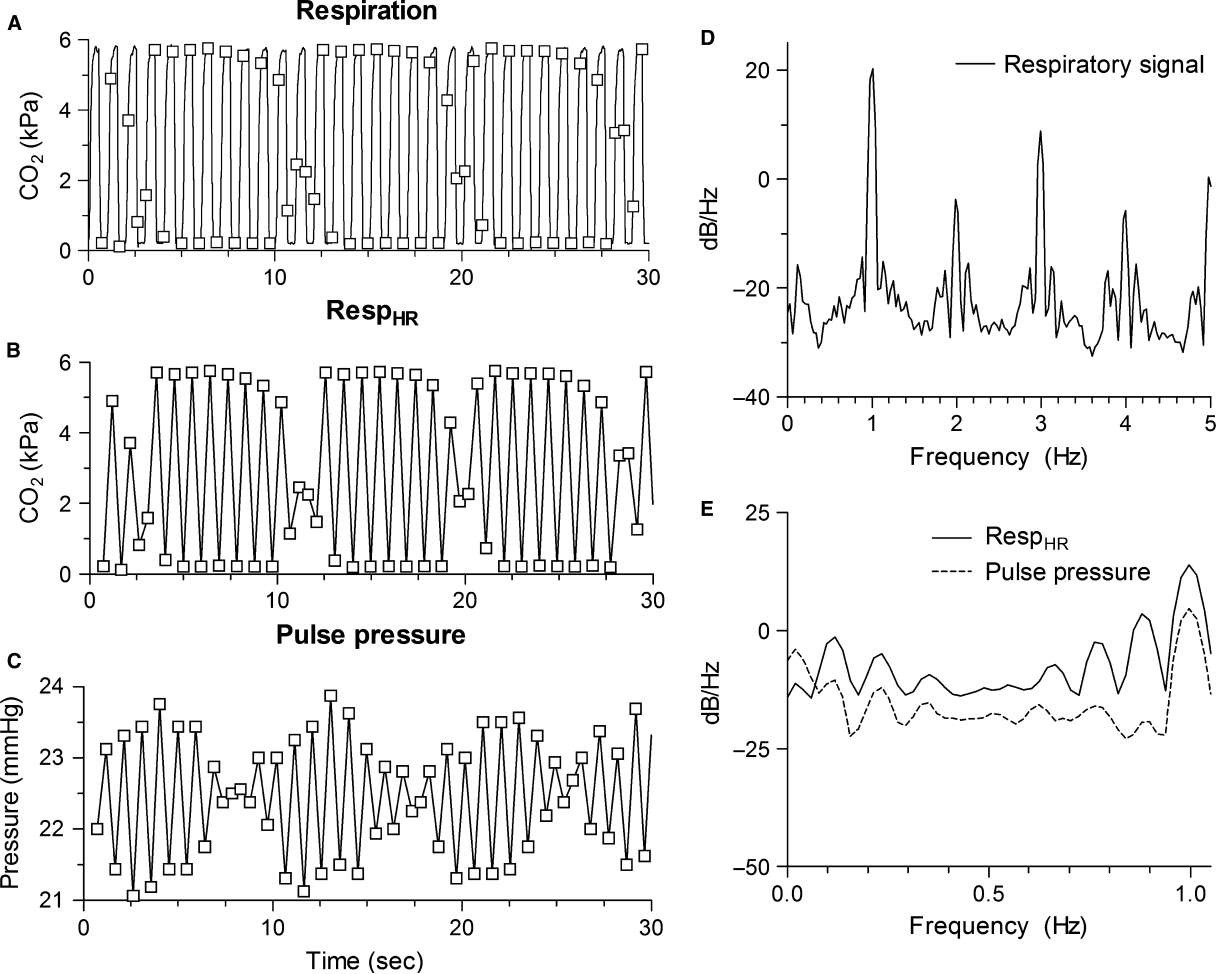
Respiratory signal, Resp_HR_, and pulse pressure in the time and frequency domain for an example patient ventilated with SIMV (RR = 1 Hz, HR = 2.1 Hz). (A) Respiratory signal with time points that systolic pressure is reached (squares). (B) Resp_HR_. (C) Pulse pressure. (D) Frequency spectrum of the respiratory signal. (E) Frequency spectra of Resp_HR_ (solid) and pulse pressure (dashed). RR, respiratory rate; HR, heart rate; Resp_HR_, the respiratory values at the moment systolic pressure was reached.

Figure 4 depicts Resp, Resp_HR_, and PP in the time domain and frequency domain for a patient ventilated with SIMV to illustrate the results (RR = 1 Hz, HR = 2.1 Hz). First, in the time domain it is seen that PP varies with inspiration and expiration as expected (Fig. 4C). In the frequency domain it is observed that this variation coincides with RR (Fig. 4D and E). Second, waves with lower frequencies are observed in PP that is also observed in Resp_HR_ (Fig. 4B, 4C and 4E). These additional lower frequencies equal the alias frequencies of the harmonic frequencies of RR. Third, a variation in PP with a frequency <0.1 Hz is observed that is not observed in Resp_HR_ (Mayer waves) (Fig. 4E). The findings illustrated in Figure 4 are observed in all patients on SIMV.

#### Relation between ventilation and PPV

The Spearman correlation between Resp_HR_ and PP was −0.64 ± 0.18. The coherence value between Resp_HR_ and PP at RR was 0.95 ± 0.11. Coherence was significant in all patients.

## Discussion

Our study shows that in neonates PPV can be observed during both SIMV and HFV and that it is related to cardiopulmonary interaction. In neonates on HFV it is observed that the frequency with which PP varies, coincidences with the alias frequency of RR. In neonates ventilated with SIMV the frequencies in PP were equal to RR or the alias frequencies of the multiples of RR. These findings support the hypothesis that the mismatch observed between RR and the frequencies in PP are caused by the low HR/RR ratio (2.6 ± 0.5 and 0.4 ± 0.1 for neonates on SIMV and HFV, respectively).

#### Effect of low HR/RR ratio

**Figure 5:**
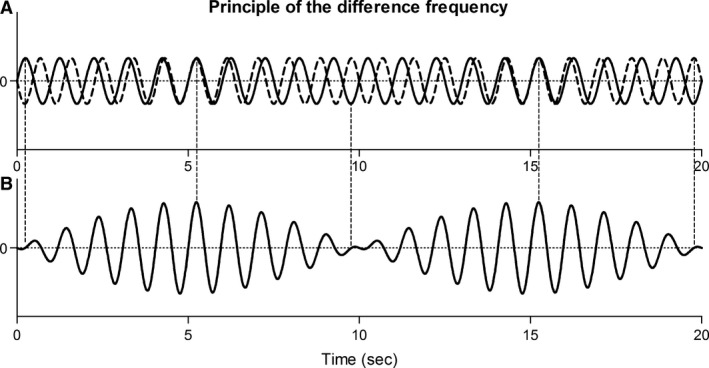
(A) Interference between two sinusoidal waveforms [1 Hz (solid), 1.1 Hz (dashed)]. (B) The difference frequency (0.1 Hz) is observed in the resulting sinusoidal waveform. This figure is a simplification of the reality.

The finding that the PP varies with the alias frequencies observed in Resp_HR_ suggests that PPV in neonates is caused by physiological aliasing. Rother et al. (1989) and Witte et al. (1988) already described this phenomenon during heart rate variability analysis. However, when a closer look is taken at the origin of PPV it is believed that not physiological aliasing, but another mechanism underlies this phenomenon. Cardiopulmonary interaction is a dynamic system that consists of the ABP with a frequency HR and the ventilation with a frequency RR, where the arterial blood pressure (ABP) is influenced by the ventilation. It is known that when RR becomes close to HR, ABP will not only vary with HR but also with a frequency equal to the difference between HR and RR, that is, the difference frequency (Pinchak et al. 1984; Mitzner et al. 1987) The occurrence of this difference frequency can be visualized by the interaction of two sinusoidal waveforms, as depicted in Figure 5 (simplification of reality). Mitzner et al. (1987) showed in a dog model that the difference frequency was observed in the pulmonary artery pressure, aorta pressure, and also in the right ventricle stroke volume. In our situation, the difference frequency equals the alias frequency, because the sampling frequency is equal to HR:

$$\begin{array}{cc} \text{Difference frequency}=|\text{HR‐RR}|\\ & \text{alias frequency}=|\text{fs}-\text{RR}|\;\text{with}\;\text{fs = HR} \end{array}$$

Interestingly, Mitzner et al. (1987) showed in their dog model that not only the difference frequency between RR and HR was observed, but also between RR and multiples of HR. Therefore, it is hypothesized that in neonates on HFV the interaction between RR and the closest multiple of HR causes the observed PPV. If this hypothesis is true, the difference frequency should already be present in the original ABP signal. If retrospectively the frequency spectrum of ABP is obtained, it is indeed observed that the difference frequency between RR and the closest multiple of HR was present in ABP. Because the original ABP signal was sampled with a frequency of 62.5 Hz and RR is always lower than half this sampling frequency (smaller than 31.25 Hz), there is no undersampling in the original ABP signal and therefore no aliasing. Hence, it is more likely that the difference frequency instead of physiological aliasing explains the presence of PPV in neonates on HFV. In neonates ventilated with SIMV it is also believed that difference frequency instead of physiological aliasing explains the frequencies in PP that did not correspond to RR. If in these patients a frequency spectrum of the original ABP signal is obtained, it is indeed observed that the frequencies in PP not equal to RR are already observed in the original ABP signal. Hence, also in neonates on SIMV it is most likely that the difference frequency explains the variation in PP at frequencies unequal to RR. During both HFV and SIMV the difference frequencies plays a role in the occurrence of the frequencies with which PP varies. However, because during HFV there is not even one heartbeat per respiratory cycle, it is possible that the underlying mechanism causing PPV is not exactly the same. The occurrence of these difference frequencies in the PP of the neonates cannot be ignored, because they influence the calculated PPV. In adults PPV is defined as the relative difference in maximal and minimal PP within one respiratory cycle. However, as clearly visible in Figure 4, the calculated PPV will depend on which respiratory cycle is used to determine PPV.

#### PPV as predictor for fluid responsiveness

PPV can be used to predict fluid responsiveness in adult patients under certain conditions (Michard and Teboul 2002; Marik et al. 2009). The finding that PPV is also observed in neonates and related to cardiopulmonary interaction supports the hypothesis that PPV might be able to predict fluid responsiveness in neonates. However, the difference in physiology between adults and neonates is of major influence on the interpretation of PPV as an indicator of fluid responsiveness. As shown in this study, the low HR/RR ratio observed in neonates results in the presence of difference frequencies, which impedes the analysis and interpretation of PPV in neonates. In adults it is already known that the predictive value of PPV is limited with a HR/RR ratio smaller than 3.6 (De Backer et al. 2005). However, they do not report the presence of the difference frequencies as observed in our study with low HR/RR ratio. In addition, other physiological characteristics might also negatively influence the predictive value of PPV in neonates. First, it is believed that in mechanically ventilated neonates the intrathoracic pressure variations are small due to a higher thoracic compliance (Papastamelos et al. 1995), ventilation with low tidal volumes (4–6 mL/kg) or ventilation with high‐frequency ventilation (HFV). In adults, it is known that the predictive value of PPV is limited with smaller intrathoracic pressure variation (De Backer et al. 2005; Muller et al. 2010; Monnet and Teboul 2013). Second, neonates have a larger arterial compliance in comparison with adults (Pereira de Souza Neto et al. 2011) that may attenuate arterial blood pressure variation and hence PPV.

Another limitation of PPV is that it can only be applied under specific conditions, as is known from adults (Monnet and Teboul 2013). PPV can only predict fluid responsiveness when patients are ventilated with positive pressure ventilation and have no spontaneous breathing activity (Heenen et al. 2006). During spontaneous breathing activity the intrathoracic pressure variation are of irregular rate and amplitude, which influences PPV. In adults, this spontaneous breathing activity is associated with loss of predictive value for PPV (Heenen et al. 2006). Lansdorp et al. (2012) showed that PPV is not able to predict fluid responsiveness in the adult ICU during routine clinical practice. In accordance with the adult ICU, at the NICU main policy is also to avoid mechanical ventilation unless it is really necessary, to keep sedation as low as possible and to promote spontaneous breathing. As a consequence, also at the NICU only a small population is mechanically ventilated without spontaneous breathing activity. It is desirable to have a technique that can be applied in a large population. Recently the passive leg raise test (Monnet and Teboul 2008; Monnet et al. 2013) and measurement of mean systemic filling pressure (Geerts et al. 2011; Maas et al. 2012) are proposed as promising techniques for predicting fluid responsiveness in critically ill adult patients. It will be interesting to investigate the applicability of these techniques in neonates. The passive leg raising test has already been investigated in children and could predict fluid responsiveness with a sensitivity of 55% and specificity of 85% (Lukito et al. 2012). However, this was not in a neonatal population (median age 6 years).

### Limitations

In this study only a small population was studied. Nevertheless, because the population varied widely in gestational age, postnatal age, and underlying pathology and the results were consistent for this widely varying population, the validity of our results for other neonates on the NICU ventilated with SIMV or HFV is supported. Furthermore, no beat‐to‐beat measurement of SV was performed. Therefore, it cannot be validated whether the variation in PP at the difference frequencies is the results of variation in SV at the difference frequencies or if there is another underlying mechanism. A third limitation of this study is that we did not determine the predictive value of PPV for fluid responsiveness in neonates. Therefore, the effect of the observed difference frequencies on the predictive value of PPV for fluid responsiveness in the newborn infant ventilated with SIMV or HFV is unknown.

## Conclusions

The observed PPV in neonates ventilated with the two most common modes of ventilation on NICU, SIMV, and HFV, is related to positive pressure ventilation. This supports the possibility to use PPV as a predictor of fluid responsiveness in neonates. However, the occurrence of the difference frequency in PP impedes the analysis of PPV. Therefore, it is doubtful whether PPV will be a reliable predictor of fluid responsiveness in neonates. Hence, it is recommended to focus further research on other indicators of fluid responsiveness.

## Conflict of Interest

The authors declare no conflict of interest.
